# Exploring nutraceutical approaches linking metabolic syndrome and cognitive impairment

**DOI:** 10.1016/j.isci.2025.111848

**Published:** 2025-01-23

**Authors:** Rebecca Sonnino, Gea Ciccarelli, Simona Moffa, Laura Soldovieri, Gianfranco Di Giuseppe, Michela Brunetti, Francesca Cinti, Eleonora Di Piazza, Antonio Gasbarrini, Enrico C. Nista, Alfredo Pontecorvi, Andrea Giaccari, Teresa Mezza

**Affiliations:** 1 Department of Translational Medicine and Surgery, Università Cattolica del Sacro Cuore, Rome, Italy; 2 Center for Endocrine and Metabolic Diseases, Fondazione Policlinico Universitario A. Gemelli IRCCS, Rome, Italy; 3 Pancreas Unit, CEMAD Digestive Diseases Center, Internal Medicine and Gastroenterology Unit, Fondazione Policlinico Universitario Gemelli IRCCS, Rome, Italy

**Keywords:** Cognitive neuroscience, Human metabolism

## Abstract

Metabolic syndrome (MetS) and mild cognitive impairment (MCI) are interconnected conditions sharing common pathological pathways, such as inflammation and oxidative stress, leading to the concept of “metabolic-cognitive syndrome.” This highlights their mutual influence and potential overlapping therapeutic strategies. Although lifestyle modifications remain essential, nutraceutical supplementation has emerged as a promising adjunct for the prevention and management of these preclinical conditions. This review examines clinical and translational evidence on commonly used nutraceuticals targeting shared pathophysiological mechanisms of MetS and MCI. By addressing inflammation, oxidative stress, and metabolic dysfunction, these supplements may offer a valuable approach to mitigating the progression and consequences of both conditions. Understanding their efficacy could provide practical tools to complement lifestyle changes, offering a more comprehensive strategy for managing metabolic-cognitive syndrome.

## Graphical abstract

**Figure:**
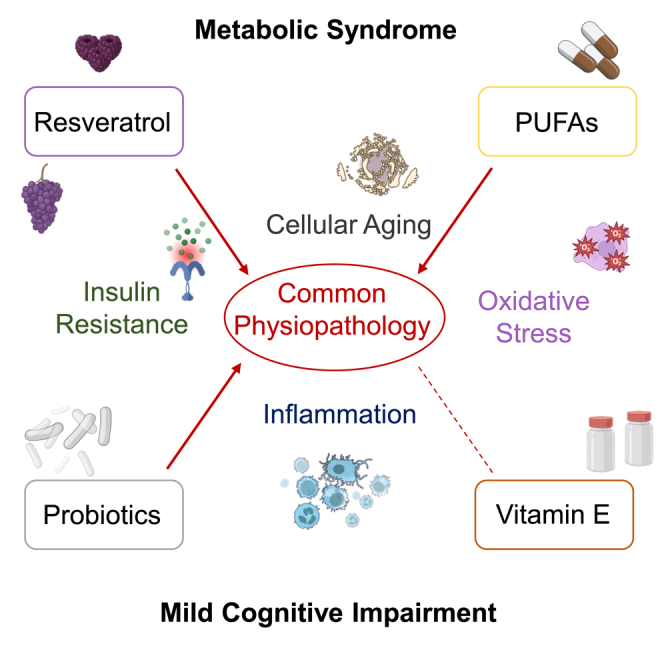

## Introduction

Morbidity and mortality due to metabolic syndrome (MetS) make it a global health concern since it is well known that, if left untreated, MetS is strongly associated with increased risk of developing type 2 diabetes mellitus (T2DM) and cardiovascular diseases (CVDs).^1^^,^^2^

Poor cognitive function is one of the many adverse health outcomes deriving from MetS. MetS is in fact associated with a higher risk of mild cognitive impairment (MCI) and the progression from MCI to dementia, particularly vascular dementia^3^ as evidenced by many cross sectional studies and systematic reviews.^4^^,^^5^^,^^6^ Not surprisingly, an inverse correlation between the presence of MetS and global cognitive performance has often been highlighted,^3^^,^^7^ and of all MetS components, high blood pressure and hyperglycaemia seem to be the strongest predictors of impaired cognitive function.^8^

One of the most investigated pathogenetic features of MetS is systemic inflammation also known as “chronic low-grade inflammation,” primarily resulting from adipose tissue dysfunction in the context of a high-calorie diet.^9^^,^^10^^,^^11^^,^^12^ This persistent inflammatory state is believed to impact brain function, representing a shared pathological substrate between MetS and MCI.^13^^,^^14^ Notably, obesity has been linked to neuroinflammation, initially observed in the hypothalamus and later identified in other brain regions such as the hippocampus, cortex, brainstem, and amygdala. This phenomenon, termed “obesity-derived neuroinflammation,” is associated with mood alterations and cognitive decline.^15^ In parallel with adipose tissue dysfunction, neuroinflammation in the brain has been linked to insulin resistance (IR), defined as reduced cellular sensitivity to insulin.^16^ More than two decades ago, research demonstrated that the brain is highly responsive to insulin, as evidenced by the presence of insulin receptors on both neurons and glial cells, circulating insulin within the brain, and localized insulin synthesis.^17^ Subsequent studies revealed a decline in the quantity and activity of insulin receptors in the brain, particularly in aging and Alzheimer’s disease (AD) models. This finding has fueled the hypothesis that neuronal IR may significantly contribute to memory impairment in these conditions.^17^^,^^18^

Some authors propose that AD and MCI could be conceptualized as an “insulin resistance brain state.”^1^ Impaired insulin signaling disrupts critical pathways essential for neuronal health and cognitive function. For example, IR diminishes the activity of the PI3K/Akt pathway, leading to aberrant activation of glycogen synthase kinase-3 beta (GSK3β). This upregulation promotes tau protein hyperphosphorylation, a hallmark of AD. Furthermore, IR interferes with O-GlcNAcylation, a modification inversely related to phosphorylation, exacerbating tau pathology. Normal insulin signaling also supports the transcription of anti-amyloidogenic proteins such as the insulin-degrading enzyme (IDE) and α-secretase, which reduce amyloid-beta (Aβ) production. When IR occurs, Aβ production and aggregation increase, triggering synaptic toxicity. Elevated Aβ levels further impair insulin signaling, creating a vicious cycle that accelerates cognitive decline.^19^

Given the strong correlation between cognitive performance and brain IR, antidiabetic drugs aimed at enhancing insulin signaling have been explored as therapeutic interventions, yielding promising results.^20^^,^^21^ Clinical guidelines for diabetes management have increasingly recognized cognitive impairment as a chronic complication of T2DM, with its incidence being approximately twice as high in individuals with T2DM compared to those without.^22^^,^^23^

The primary cause of the interesting overlap between MetS and neurodegenerative disorders is still a matter of debate. However, due to the emerging awareness of this intricate link between metabolic features and cognitive decline, the concept of “metabolic-cognitive syndrome” has recently been introduced indicating a cross-talk between the pathological mechanisms involved in both conditions and their propensity to occur together.^8^

Hyperglycemia and IR are known to drive the overproduction of reactive oxygen species (ROS), neuroinflammation, and the disruption of key signaling pathways such as the PI3K/Akt pathway. These changes lead to cellular damage in critical brain regions like the hippocampus and hypothalamus, which play essential roles in cognitive processes, learning, and memory. Alterations in the hippocampus are also linked to deficits in executive functions, such as reduced cognitive flexibility and increased impulsivity. These impairments can hinder effective weight management and adherence to dietary regimens, thus perpetuating the interdependence of MCI and MetS within a complex and self-reinforcing vicious cycle.

Conversely, weight loss has been consistently associated with improvements in cognitive functions, including memory and executive control. Supporting this connection, several studies have explored dietary interventions using functional food products, such as *Opuntia ficus-indica* (prickly pear cactus), in animal models of MetS. These studies reported enhanced behavioral performance and overall cognitive improvements. At the biochemical level, such interventions were shown to improve lipid profiles and reduce oxidative stress markers, with concurrent modulation of circulating leptin levels.^24^ Further evidence from mouse models of MetS-associated cognitive decline highlights the benefits of dietary changes. Specifically, transitioning from a high-fat diet (HFD) to a low-fat diet and administering repeated infusions of plasma from healthy mice to HFD-fed mice have demonstrated notable memory improvements. These findings emphasize the potential of dietary strategies in mitigating cognitive decline linked to MetS.^25^

As both MetS and MCI are characterized by a slow and discontinuous progression, they represent an intermediate clinical phenotype between a healthy condition and the fully manifest disease. Therefore, both disorders can be seen as a time interval in which it may be possible to intervene and delay progression to dementia^23^ or T2DM and cardiovascular outcomes.^1^ Unfortunately, this silent evolution makes the diagnostic work up slower. Moreover, given the inconclusive data available regarding the exact mechanism behind them, there are currently no codified therapies available for treatment or prevention.

Certainly though, MetS and MCI are both multifactorial conditions, with environmental and genetic factors contributing to their development. In addition, lifestyle factors, especially lack of physical activity and excessive nutritional intake, have been identified as major contributors.^1^ Consequently, multidomain lifestyle modifications have been proposed as a valid therapeutical strategy.^9^^,^^10^^,^^26^^,^^27^^,^^28^^,^^29^ Cardio metabolic risk factors usually develop in middle age, suggesting the importance of preventive approaches for target population in their 40s and 50s. Also, it has been demonstrated that young adulthood and middle age are crucial periods for determining cognitive health in old age.^30^ Modifiable lifestyle factors in the prevention of MCI and dementia are diet, physical activity, cognitive and social engagement, smoking and alcohol consumption. Similarly well-known modifiable lifestyle factors in the prevention of MetS and then T2DM are diet and physical activity. Based on the importance of diet for both conditions and on the urgency of prevention, great interest has risen in the non-pharmacological approach through the use of nutraceuticals.^31^^,^^32^^,^^33^ Nutraceuticals are a food or part of food capable of providing beneficial health effects, including the prevention and the treatment of diseases.^34^ These products have risen interest in different fields for the fewer side effects and long-term safety along with a broad therapeutic effect. Indeed, the same nutraceutical can address different diseases with common pathways and risk factors, as MCI and MetS.

Since MCI and MetS are conditions at risk of evolution in overt non-communicable diseases—T2DM and dementia—and since a recognized pharmaceutical strategy has not been found for any of the two conditions, in this review, we aim to explore data on the nutraceutical approach to MCI and MetS, as a possible alternative to medical treatment and/or as an useful add-on to diet and physical activity. Specifically, we explore the role of different type of nutraceuticals, as vitamins, botanicals, dietary supplements and probiotics, known for their anti-inflammatory and antioxidant properties, that may exert a role in MCI and/or MetS. There is a considerable body of literature on the subject and our selection was guided mostly by the potential role of these compounds in clinical practice. Also, we did not reported literature about well-known and recognized nutraceuticals in the context of MCI and MetS, such as B group vitamins and iron, and, in contrast, we did not reported nutraceuticals that have not been studies in these two conditions so far. We rather focused on elements presenting heterogeneous results in randomized clinical trials (RCTs), highlighting gaps in the literature and possible critical explanations for any controversial finding. In Table 1, we listed the compounds that we have selected, classified according to their properties, i.e., mostly anti-inflammatory, mostly antioxidant or both.

**Table 1:** Classification of the studied compounds according to their properties

Active ingredient	Mostly anti-inflammatory	Mostly antioxidant	Both properties	Actions
Resveratrol	–	–	x	Upregulating AMP-activated protein kinase. Increase endothelial nitric oxide bioavailability. Inhibition of COX-2, 5-lipoxygenase, and nuclear factor-κB. Upregulation of IL-10 and the downregulation of interferon-γ and IL-17.
Tocotrienols (T3) and tocopherols (TF)	x	–	x	Suppressing M1 macrophage differentiation. Increase NGF levels. ↓ of the glutathione redox ratio and plasma lipid peroxidation. Vasorelaxation by stimulation of K-Ca channel
PUFAs	x	–	–	Downregulation of IL-6, tumor necrosis factor alpha (TNF- α), CRP, and IL-1 levels. Converting the phenotype of microglial cells from macrophage M1 state to an M2 state.
Probiotics	x	–	–	High IL-10-inducing activity in immune cells. Downregulation of hs-CRP and TNF-α levels

## The role of vitamin E in MetS

Vitamin E is a fat-soluble vitamin found in seeds, nuts and seed oils. Its components, particularly tocotrienols (T3) and tocopherols (TF), are antioxidants, due to their ability to utilize the free hydroxyl group on the chromanol ring to capture free radicals, resulting in a reduction of the glutathione (GSH) redox ratio and plasma lipid peroxidation.^35^^,^^36^ Due to the latter properties the effects of vitamin E, particularly T3 and TF, on MetS have been investigated in both animal models and human studies with contradictory results.^37^

In obese mouse models, Zhao et al.^38^ have shown that T3 supplementation leads to accumulation of this vitamin in adipose tissue (AT). Mice were fed a HFD supplemented with 0.05% γT3 for 4 weeks and showed a lower accumulation of fat depots in liver, epididymis, and mesentery than MetS-mice without vitamin E supplementation. Concomitantly, mice supplemented with γT3 also showed lower levels of fasting glucose, insulin and proinflammatory cytokines, improved glucose tolerance and insulin sensitivity. Zhao et al. also demonstrated *in vitro* that T3 may exert a positive role in impaired AT by suppressing M1 macrophage differentiation, reducing inflammation and ultimately improving insulin signaling.^38^ This evidence has been recently confirmed by Kato Y,^39^ who demonstrated that treatment with T3 inhibited white adipose tissue accumulation and elevation of serum cholesterol concentrations in HFD mice.

Studies in humans have yielded similar positive results: Manzella et al.^40^ observed a positive effect of vitamin E (all-rac-alpha-tocopheryl acetate, 600 mg die for 4 months) supplementation on the cardiac autonomic nervous system in subjects with diabetes, showing an increased antioxidant capacity, significantly decreased plasma catecholamine concentrations, Homeostasis Model Assessment (HOMA) index and HbA1c values. The 4 months length of time was specifically chose by the authors as they considered it the minimum length of time necessary to observe effects of vitamin E supplementation, based on previous groups’ studies.^41^ Considering oxidative stress as a main effector of the unbalanced sympathetic-parasympathetic tone typically expressed in subjects with diabetes, these results may indicate that vitamin E, which lowers oxidative stress, could exert beneficial effects by improving metabolism, downregulating inflammatory mediators and rebalancing autonomic activity. These results in diabetic models appear to be congruent with an effect of vitamin E on oxidative stress induced by glucotoxicity. Interestingly, there is also indirect evidence that this nutrient plays a role in the preservation of beta-cells.^42^^,^^43^^,^^44^ Indeed, Asayama et al.^45^ found that rats deficient in vitamin E had diminished insulin secretory reserves, implying that vitamin E level can directly influence pancreatic islet function.

Vitamin E has also been investigate-d in hypertension. Supplementation with tocotrienol-rich fraction (TRF) was found to reduce the incidence of pregnancy-induced hypertension.^46^^,^^47^ In particular, Aminuddin et al.^47^ showed an association between vitamin E (TRF 100 mg daily in super olein for a six month period minimum, beginning from 12 to 16 gestational weeks until delivery) supplementation and a reduction in the incidence of preeclampsia and maternal hypertension, after controlling for other confounders such as age and maternal weight (Table 2).

**Table 2:** Offers a view of the proposed mechanism by which some nutraceuticals may have a positive impact on MetS and MCI. It summarizes the nutraceuticals selected in this review and offers a schematic representation of possible implications for clinical practice

Efficacy	Action	References
**Vitamin E on MetS**		

Potentially useful	Reduces levels of proinflammatory cytokines. Reduces M1 macrophage differentiation. Improves glucose tolerance and insulin sensitivity	Zhao et al.^38^
Potentially useful	Decreases plasma catecholamine concentrations, HOMA index and HbA1c values	Manzella et al.^40^
Potentially useful	Influences pancreatic islet function	Asayama et al.^45^
Potentially useful	Positive impact on body weight	Wong et al.^37^
Potentially useful	Lowering total cholesterol, LDL-C, apoB, TG, FBG, and glucagon levels	Qureshi et al.^48^
Potentially useful	Anti-hypertensive effect	Aminuddin et al.^47^
Not useful	Anti-hypertensive effect	Palumbo et al.^49^

**Vitamin E on MCI**		

Potentially useful	Up- or downregulating some genes involved in the pathogenesis of neurodegeneration	Gugliandolo et al.^50^
Potentially useful	Increases expression of NGF	Rota et al.^51^
Potentially useful	Delay loss of cognition	Maxwell et al.^52^
Not useful	Delay loss of cognition	Lloret et al.^35^
Not useful	Delay loss of cognition	Morris et al.^53^
Not useful	Delay loss of cognition	Jae et al.^54^

**RVS on MetS**		

Potentially useful	Positive impact on insulin resistance and pancreatic beta-cells by the upregulation of AMP-activated protein kinase	Szkudelski and Szkudelska^55^
Potentially useful	Upregulation of lipolysis and reduction of lipogenesis in mature adipocytes	Baile et al.^56^
Potentially useful	Increase endothelial NO bioavailability, modulation of the renin-angiotensin system	Kim et al.^57^
Potentially useful	Glycemic control and insulin sensitivity, along with a reduction in serum levels of inflammatory markers	Timmers et al.^58^
Not useful	Increasing HDL-C level, decreasing BMI, TG, and FBG	Asgary et al.^59^

**RVS on MCI**		

Potentially useful	Neuroprotective effect by upregulation of neurogenesis and microvasculature, downregulation of astrocyte hypertrophy and microglial activation in the hippocampus	Zhou et al.^60^
Potentially useful	improvement in spatial and emotional learning memory	Gocmez et al.^61^
Potentially useful	Vasorelaxation by stimulation of K-Ca channel	Li et al.^62^
Potentially useful	Improvement in verbal memory performance by increasing functional connectivity of the hippocampus and reduction of glucotoxicity in brain tissue	Veronica Witte et al.^63^

**PUFAs on MetS**		

Potentially useful	Effect on BP and inflammatory markers such as IL-6, TNF-α, CRP, and IL-1 levels	Wang et al.^64^
Potentially useful	FBG control by insulin sensitivity, BP	Liu et al.^65^
Potentially useful	FBG control	Belchior et al.^66^
Potentially useful	Reduction the levels of brain receptor for advanced 14 glycation end-product and plasma TNF-α	Y. R. Guo et al.^67^
Not useful	FBG control	Simão et al.^68^
Not useful	FBG control	Mori et al.^69^

**PUFAs on MCI**		

Potentially useful	Modulation of the production of inflammatory cytokines. Upregulation of antioxidant proteins, conversion of microglia cell phenotype from macrophage M1 state to an M2 state	Serini and Calviello^70^
Potentially useful	Impact on MMSE	Freund-Levi et al.^71^
Potentially useful	Impact on MMSE	Lee, Shahar, Rajab et al.^72^
Potentially useful	Impact on MMSE	Sinn et al.^73^
Potentially useful	Impact on MMSE	Mazereeuw et al.^74^
Not useful	Impact on MMSE	Solfrizzi et al.^75^

**Probiotics on MetS**		

Potentially useful	Improvement in waist circumference and in fasting plasma FBI, lipidic profile	Cicero et al.^76^
Potentially useful	Significantly lower total cholesterol and LDL-C	Z. Guo et al.^77^
Not useful	Impact on HDL-C and triglycerides	Z. Guo et al.^77^

**Probiotics on MCI**		

Potentially useful	Improvement in cognitive function associated with lower level of MDA and hs-CRP	Den H et al.^78^
Potentially useful	Suppressed brain atrophy progression	Asaoka et al.^79^
Potentially useful	Suppressed the hippocampal expressions of inflammation	Xiao et al.^80^
Potentially useful	Improvement of cognitive functions as measured with RBANS total score associated with HbA1c reduction	Bernier et al.^81^
Potentially useful	Visual memory: gut microbiota, supposed correlation with blood ammonia levels and IL-1 activity	Sakurai et al.^82^
Not useful	No differences observed in “immediate memory”	Kobayashi et al.^83^

BMI, body mass index; BP, blood pressure; CRP, C-reactive protein; FBG, fasting blood glucose; FBI, fasting blood insulin; HbA1c, glycated hemoglobin; HDL-C, high-density lipoprotein cholesterol; HOMA-IR, homeostasis model assessment of insulin resistance; IL-1, interleukin 1; IL-6, interleukin 6; LDL-C, low-density lipoprotein; NGF, the nerve growth factor; NO, nitric oxide; TNF-α, tumor necrosis factor alpha.

Other studies aiming to assess the effect of antioxidant supplementation on hypertension, have found a hypotensive effect.^84^^,^^85^ Following the same rationale, many RCTs have tried to demonstrate a beneficial effect of vitamin E on hypertension, with conflicting results. Palumbo et al.^49^ explored the effect of vitamin E (300 mg once a day for 12 weeks), on systolic and diastolic blood pressure in treated hypertensive patients. The authors found no clinically relevant effect on blood pressure in hypertensive patients already in treatment. The same results were obtained in a study^86^ on 89 randomized patients treated with vitamin E 1,800 international units (IU) daily or placebo for a 12 month-period (Table 2).

Despite these contradictory results, vitamin E has proven overall to have a positive impact on the oxidative stress and systemic inflammation generated by metabolic conditions such as MetS. However, more research is required to assess the exact target of this nutraceutical, in order to standardize it as a useful tool for MetS treatment.

## The role of vitamin E in MCI

As a correlation has been reported between AD and MCI and lower levels of tocopherol,^87^ many studies have investigated the antioxidant properties of vitamin E and its major elements for a possible role in lowering or delaying the process of intellectual deterioration in MCI^88^ with conflicting results.

Studies exploring the role of vitamin E on cognitive impairment in both animal models and human patients^50^^,^^89^ found it useful not only for its antioxidant capacity, but also because it has an up- or downregulating effect on some genes involved in the pathogenesis of neurodegeneration in AD. Indeed, vitamin E deficiency has been shown to induce an increase in cerebellar oxidative stress, as suggested by the high levels of protein nitrosylation found in a Ttpa−/− knock-out mouse model.^50^ The Ttpa gene encodes for a protein responsible for the selective excretion of RRR-α-tocopherol from hepatocytes to circulating lipoproteins that transfer the vitamin to non-hepatic tissues, such as neurological tissue. Subjects carrying loss-of-function mutations in the Ttpa gene develop ataxia associated with extremely low vitamin E levels (less than 2 μM) compared with the normal range of 18–36 μM.^90^ Furthermore, a significant expression of Ttp was demonstrated in the brain as well as in the placenta, suggesting that this protein provides additional protection to vulnerable tissues.^50^^,^^90^

Moreover, comparing rats receiving a diet without vitamin E with rats fed a standard diet, Rota et al.^51^ found that the nerve growth factor (NGF), a neurotrophin that promotes neuron survival, is less expressed in rats without vitamin E supplementation, compared with models supplemented with 60 mg/kg for a period of 9 months.^51^ In contrast, studies investigating the efficacy of vitamin E supplements to treat MCI and AD have found no evidence that this intervention prevents progression of dementia or improves overall cognitive function.^49^^,^^90^^,^^91^

Some authors^35^ have explained the contradictory results on the efficacy of vitamin E in improving cognitive function with the interesting concept of “the vitamin E paradox”: in a prospective, double blind, placebo-controlled study with 57 AD subjects, 800 IU of vitamin E supplementation per day for six months led to an acceleration in mental deterioration. Studies have found a possible explanation for these results in identifying two sub populations who react in opposite ways to vitamin E supplementation: subjects in whom vitamin E acts as an antioxidant, respond positively to treatment, especially in terms of prevention of functional decline, while in the other subset of patients, who do not respond to treatment with this vitamin, there is no improvement in oxidant level, with a worsening in cognitive function^91^^,^^92^ (Table 2). However, the clinical traits that distinguish the different phenotypes remain as yet unknown.

## The role of vitamin E in the prevention of MCI/MetS

A limited number of studies have examined the effects of vitamin E on cognitive function in healthy populations, with inconclusive and conflicting results.^93^ Maxwell et al.^52^ using data from the Canadian Study of Health and Aging (CSHA), reported that supplemental use of antioxidant vitamins, including vitamin E, reduced the risk of significant cognitive decline and the incidence of vascular dementia.

The prospective Rush Memory and Aging Project^53^ showed an inverse association between levels of γ-tocopherol and amyloid loads along with neurofibrillary tangle pathology. However, the trial failed to provide evidence of a statistical interaction between clinical cognitive impairments and tocopherol levels. On the contrary, the Women’s Health Study, with 6,377 women over 65 years, observed no significant effect of long term use of vitamin E on cognitive function.^54^ In a phenytoin-induced cognitive impairment rat model, Nagib MM and colleagues demonstrated that alpha tocopherol supplementation alleviates the cognitive impairment suppressing oxidative damage.^94^ Also, in old rats, vitamin E have proved to improve cognitive and motor abilities, through increasing the synthesis rate of monoaminergic neurotransmitters.^95^

This finding suggests the possible preventive function of vitamin E on cognitive impairment; however, more RCTs with selective use of vitamin E are required to confirm this.

Vitamin E has also been investigated for its anti-hyperlipidemic properties, through post-transcriptional mechanisms. In healthy animal models, studies have shown a reduction in cholesterol and a significant effect in decreasing body weight. Furthermore, Qureshi et al.^48^ showed efficacy of 50 mg alpha-tocopherol per day for 35 days (compared with lovastatin) in lowering total cholesterol, LDL-C, apoB, triglycerides (TG), glucose, and glucagon levels in a hereditary hypercholesterolemic swine model.

Vitamin E has also been reported to have an inverse association with non-alcoholic fatty liver disease (NAFLD) in humans. Qi et al.^96^ conducted a cross-sectional study with data from the National Health and Nutrition Examination Survey (2017–2020), and after adjusting results for confounders, observed that dietary intake of vitamin E had a protective role against NAFLD^48^^,^^97^ (Table 2).

Kato Y and colleagues^98^ have demonstrated—in HFD mice—that T3 treatment inhibits body weight gain and attenuates brain oxidation. It seems that vitamin E can delay but not arrest the body weight gain associated to a hypercaloric diet and it can prevent brain oxidation via antioxidative effect as well as by upregulating antioxidative defense system.^98^ Later, the same group has demonstrated that high fat high sucrose diet in mice decreases their learning ability and that T3 treatment prevents its change.^99^ All this evidence show how vitamin E could prevent first steps of obesity-related cognitive impairment.

These findings could suggest a potential role of vitamin E in the prevention of MetS, as some studies have highlighted the impact of this nutrient on all syndrome-related comorbidities.

## The role of resveratrol in MetS

Resveratrol (RSV), known also as 3,5,4V-hydroxystilbene, is a natural polyphenol produced by plants in response to environmental stress.^100^ As all polyphenols, it derives from phenylalanine and is composed of an aromatic ring with a reactive hydroxyl group.^101^ It is a popular nutraceutical, known especially for being responsible for the beneficial health effects of wine, and identified as the main player in the questionable “French paradox.”^100^ Foods containing this extract, such as cocoa, green tea and wine, are derived from plant sources.^102^

Scientific research has investigated this element for its involvement in various pathways, and specifically for its ability in targeting antioxidant and anti-inflammatory mediators.

Due to its pleiotropic effect RSV has a substantial impact on metabolism, as evidenced in animal and human studies. It has been shown to improve glucose homeostasis and insulin secretion, playing a role in the modulation of IR, and protecting pancreatic beta-cells mainly by upregulating adenosine monophosphate (AMP)-activated protein kinase in various tissues of diabetic subjects.^55^

RSV has been shown to improve body fat accumulation and energy balance. In both white and brown adipose tissue it actively decreases lipid accumulation by modulating different pathways resulting in upregulation of lipolysis and reduction of lipogenesis in mature adipocytes.^56^^,^^103^

Considering the crucial role played by oxidative stress in the development of vascular dysfunction triggered by hypertension, RSV has been shown to increase endothelial nitric oxide (NO) bioavailability by enhancing its transduction pathway. Kim et al.^57^ have shown a positive effect of a mixture of RSV 40 mg/kg for 6 months on vascular dynamics, and thus on hypertension, through the modulation of the renin-angiotensin system. However, whether RSV has an effect on lowering blood pressure or not, remains debatable.^104^

Asgary et al.^59^ carried out a meta-analysis to determine the association between resveratrol intake and metabolic parameters in patients with MetS. Results were encouraging and consistent with a positive impact of nutraceuticals on MetS. Out of 16 studies analyzed, (10 on humans and 6 on animal models), supplementation with RSV at the dosage of >500 mg proved to be effective in increasing high-density lipoprotein (HDL) levels, and decreasing BMI, TG, and glucose levels^59^ (Table 2).

Due to its impact on all MetS domains this nutrient is therefore an excellent candidate for MetS treatment and prevention.

## The role of RSV on MCI

There is an ample body of literature documenting the beneficial effects of RSV on cognitive decline.^60^^,^^105^^,^^106^^,^^107^ In animal models, RSV treatment improved memory and learning ability compared with controls. Resveratrol also improved net neurogenesis and microvasculature, decreased astrocyte hypertrophy, and microglial activation in the hippocampus, demonstrating an effective neuroprotection.^60^ *In vivo* experiments conducted by Gocmez et al.^107^ on both normal aging and cognitively impaired animal models, showed that RSV 50 mg/kg/day for 12 weeks could reverse cognitive impairment via inhibition of the production of inflammatory cytokines resulting in an improvement in spatial and emotional learning memory.^107^

The most studied mechanism through which RSV is believed to provide neuroprotection is its ability to reduce some proinflammation pathways, through the inhibition of the cyclooxygenase-2 (COX-2), 5-lipoxygenase, and nuclear factor-κB. It is also associated with the upregulation of interleukin (IL)-10 and the downregulation of interferon-γ and IL-17. Both anti-inflammatory and antioxidant effects of this natural component represent an attractive alternative in the treatment of neurodegenerative diseases.^105^ Moreover, it has been shown that in human endothelial cells, resveratrol induced vasorelaxation by stimulation of K-Ca channel^62^ that could be the mechanism responsible for its preventive role in CVD and their consequences.

Experimentation with RSV supplementation in normal aging humans has shown ubiquitarian effects. Witte et al.^63^ reported that 26 weeks of resveratrol intake (200 mg/d) resulted in improvement of verbal memory performance through an increased functional connectivity of the hippocampus; moreover, subjects in the treatment group showed a significant reduction in HbA1c compared with placebo.^63^ This double-positive effect was confirmed in MCI human models: an RCT performed on 110 subjects diagnosed with MCI^106^ showed a moderate reduction of HbA1c concentration and preserved left anterior hippocampus volume. These results seem to be in line with the previously demonstrated effect of RSV on cerebral flow. The authors also describe the reduction of glucotoxicity in the brain tissue and the increase in the functional connectivity between the hippocampus and different brain regions as the potential mechanisms underlying the positive influence of RSV on neuroactivity. As reviewed by Buglio et al.^105^ literature offers many examples of RCTs showing the benefits of RSV in patients with MCI or AD. Moreover, RSV has been shown to have a preventive effect on AD, reducing amyloid plaques in animal models with AD^107^ (Table 2).

These promising results highlight a possible therapeutic strategy for MCI, as there is currently no effective pharmacological intervention for this condition.

## The role of RSV in the prevention of MCI/MetS

RSV has been reported to play a protective role in hypertension, dyslipidaemia, obesity, and IR. It also been shown to act as an antioxidant and to be neuroprotective by inhibiting the production of inflammatory cytokines.^60^^,^^105^^,^^108^^,^^109^ Therefore, given the possible benefits in a healthy population, including weight loss, rebalanced metabolic function and normal aging, RSV emerges as a promising tool for the prevention of MetS and MCI.

In female senescence accelerated mouse-prone mice, it has been demonstrated that a resveratrol-enriched diet, for two months prior to mating, prevent cognitive impairment in mice offspring through epigenetic changes and cell signaling pathways.^110^ Moreover, Wu Z and colleagues^111^ have demonstrated that a resveratrol and selenium rich milk supplementation ameliorates cognitive function in d-galactose-induced cognitive dysfunction model mice.^111^

In obese humans, Timmers et al.^58^ reported a variety of improvements after treatment with 150 mg per day of RSV for 30 days, including glycemic control and insulin sensitivity, along with a reduction in serum levels of inflammatory markers. Konings et al.,^112^ on the other hand, focused on changes in adipose tissue morphology, showing a reduction in the size of abdominal subcutaneous adipocytes in subjects treated with RSV (150 mg per day) for 30 days (Table 2).

Regarding its role in MCI prevention, as previously reported, Witte et al.^63^ proved its effectiveness in neuroprotection in normal subjects. The study analyzed the effect of 200 mg/d of RSV for 26 weeks in twenty-three healthy overweight older patients. Through the assessment of working memory, neuroimaging, serum parameters, anthropometric records, and vascular markers, the authors registered improvements in the integrity and functionality of the hippocampus, along with a reduction in body fat and in HbA1c.

Experimental findings have proved RSV to be effective, to some extent, in both metabolism and neuroactivity pathways. Indeed, Gocmez and collaborators have demonstrated that resveratrol supplementation (20 mg/kg/day, intraperitoneally [i.p.]) for 4 weeks after induction of diabetes by streptozotocin in rat prevented cognitive decline, occurring in diabetes mice without resveratrol supplementation. In particular, their data demonstrated that resveratrol prevents memory deficit, endothelial dysfunction, increased oxidative stress and inflammation in a vascular dementia rat model.^61^^,^^107^ A similar result has been shown by Tian and colleagues, who demonstrated that resveratrol supplementation improved neuronal injury and cognitive performance by attenuating oxidative stress and inflammation as well as inhibiting synapse loss in diabetic rats.^113^

Overall, this evidence demonstrated that resveratrol may be useful in MetS and MCI alone, but also to prevent cognitive decline in metabolic disease.

## The role of PUFAs on MetS

Dietary intake of polyunsaturated fatty acids (PUFAs) and omega-3-fatty acids has been shown to have positive cardiovascular effects. Major sources of PUFA are tofu, and some nuts and seeds such as walnuts and sunflower seeds, while omega-3-fatty acids, eicosapentaenoic acid (EPA, 20:5), and docosahexaenoic acid (DHA, 22:6), are mostly found in fish and other seafood. It is well known that dietary patterns enriched with these foods are associated with positive cardiometabolic outcomes.^114^ For this reason this supplement is largely used in clinical practice, as omega-3-fatty acids in particular are the first-line therapy for hypertriglyceridemia.^66^^,^^115^

A meta-analysis was run in 2023^64^ on the effects of omega-3 PUFAs on the different features of MetS, such as total lipid profiles, blood pressure, and inflammatory markers. As a result, the authors confirmed a significant reduction in TG levels, with a linear relationship between the duration of omega-3 PUFAs intake and changes in TG. Interestingly, supplementation also had a favorable effect on blood pressure and inflammatory markers such as IL-6, tumor necrosis factor alpha (TNF-α), C-reactive protein (CRP), and IL-1 levels.

A review by Brady et al.^116^ on the intake of very long chain PUFAs (VLC *n*-3 PUFA) in MetS subjects found similar effects on lipid profiles, but no impact on glucose metabolism and insulin action. Regarding the effect on glycemic metabolism, some studies^68^^,^^69^^,^^117^ highlighted a worsened glucose control in the intervention group, while others^113^^,^^118^ evidenced a protective role. A recent study suggested that use of *n*-3 PUFAs supplements may significantly decrease serum triglyceride levels and lower blood pressure in patients with MetS. In this study, *n*-3 PUFAs increased insulin sensitivity, leading the authors to hypothesize a role in the prevention of hyperglycaemia^65^ (Table 2).

## The role of PUFAs on MCI

The composition of the neuronal membrane is believed to be directly affected by diet; this idea has focused attention on the role of PUFA supplementation on brain health,^119^ including psychological distress^120^ and cognitive impairment. PUFAs influence cognitive function through several mechanisms, with effects on brain development, neurotransmission, modulation of ion channels, and moreover neuroprotection.^119^^,^^121^^,^^122^ Additionally, many of these functions are dependent on neuronal membrane fluidity; as rigidity increases during aging, PUFA are believed to be useful in maintaining the proper architecture of the membrane.^123^

One of the interesting aspects of PUFAs is their ability to modulate the production of inflammatory cytokines,^70^ which are supposed to be active effectors of neurodegeneration, in different pathological conditions. Data from preclinical studies support the hypothesis that PUFAs can actively interfere with inflammatory pathways involved in neurological weakening by positively regulating the transcription of nuclear erythroid 2-related factor 2 (Nrf2), resulting in an upregulation of antioxidant proteins, and by converting the phenotype of microglial cells from macrophage M1 state to an M2 state.^70^

Several RCTs^71^^,^^72^^,^^73^^,^^124^ have been conducted with the aim of proving that regular intake of these micronutrients can help the neurological system of subjects with MCI better cope with aging and its inflammatory pattern.

Freund Levy et al.^71^ performed an RCT analyzing the effect of six-month supplementation of ω-3 fatty acids (1.7 g of DHA and 0.6 g of EPA) in patients with mild to moderate AD and found no significant impact as measured using the mini-mental state examination (MMSE) or the cognitive portion of the AD assessment scale. Interestingly, the study of a subgroup of 32 patients with very mild AD (MMSE 27 points) revealed a statistically significant treatment effect in the MMSE scores over time between the 2 groups. The authors concluded that when the disease is clinically advanced, the neuropathological substrate is too developed to be attenuated by anti-inflammatory activity. As shown by this trial, there may be a critical period to halt this process, i.e., 2 years or more before the onset of dementia, during which inflammatory molecules likely mediate cognitive impairment and can be successfully targeted to restore cognitive function.

To explore this possibility, Solfrizzi et al.^75^ conducted a prospective study on non-demented elderly subjects to evaluate the role of PUFA supplementation. Their data revealed that only high-dose PUFA supplementation showed a borderline, but non-significant, trend for a protective effect against the development of MCI.

In subjects with MCI, a 12-month period intervention with fish oil supplementation high in DHA (3 soft gelatin capsules each day, each containing 430 mg of DHA and 150 mg of EPA), showed a positive impact on almost every cognitive domain.^72^ This evidence supports the hypothesis that MCI is an intermediate phase in the cognitive decline spectrum, which can be reversed if properly treated. Promising results on cognitive domains have been observed in a cohort of 50 people aged over 65 with MCI randomized to receive a supplement rich in EPA (1.67 g EPA þ 0.16 g DHA/d; *n* 17), DHA (1.55 g DHA þ 0.40 g EPA/d; *n* 18), or the *n*-6 PUFA linoleic acid (LA; 2.2 g/d; *n* 15) for 6 months,^73^ although no significant effect was reported overall on depressive symptoms and quality of life. A meta-analysis by Mazereeuw et al.^74^ also reported a protective effect on certain cognitive domains in MCI subjects after supplementation with *n*-3 fatty acids.

This benefit on selected neuropsychological spheres may be due to targeted modulation of synaptic plasticity in specific brain areas that are particularly susceptible to oxidative stress.^125^

Similar results were obtained in a case control study run in 2013 by Lee et al.^126^ in which PUFA intake was found to be positively correlated with cognitive function in patients with MCI. In this study, lipid peroxidation was used as a biomarker of oxidative damage as it is known to be higher in MCI patients compared with the normal aging population, and was found to be significantly decreased after regular administration of PUFAs compared to the control group (Table 2).

These studies do, however, have some limitations, for example, the length of the follow up, limited to up to six months, and the small size of the cohort analyzed. Moreover, considering the countless biological pathways involved in neuronal aging, it is difficult to decide which markers should be selected for this kind of research.

Overall, in RCTs, PUFAs have proven to have some influence on neural development, neurological aging, neuroinflammation and neuroprotection in general,^127^ together with a high safety and tolerability profile. Therefore, the introduction of PUFAs supplementation is probably a promising alternative in the management of MCI.

## The role of PUFAs in the prevention of MCI/MetS

PUFAs interventions in MCI in prediabetic, non-cognitively impaired animal models have shown protective effects on brain health.^67^ In fact, Guo et al. showed that brain aging models (induced by D-Gal administration), with metabolism-related disorders, benefited from dietary intake of PUFAs. Neurocognitive tests and biochemical essays to detect metabolism parameters and oxidant activity showed that PUFAs exerted two effects: a reduction in the levels of brain receptor for advanced 14 glycation end-product (marker of oxidative stress) and plasma TNF-α, and the attenuation of abnormal metabolic characteristics in the D-gal-treated aging rats.^67^

In humans, data from a meta-analysis^74^ investigating the impact of PUFAs on cognitive health, showed the lack of accountability across these studies due to several biases, such as the short intervention period and the authors ability to statistically detect the potential beneficial effect of PUFAs on cognitive function in a healthy aging population^128^^,^^129^^,^^130^ (Table 2). However, given the positive effects found in cognitively impaired patients, the potential of a protective role of PUFAs remains intriguing and should be further investigated with RCTs to fill this gap in the literature.

Given the importance of the MetS in the genesis of CVD, and the pivotal role of hypolipidemic therapy in its prevention, it seemed reasonable to investigate the association of PUFA with the risk of coronary heart disease and major cardiovascular events. A meta-analysis published in 2017^131^ examined ten trials with a total of 77,917 patients and found neither a positive nor a negative association between omega-3 fatty acid supplementation and cardiovascular events.

Preclinical studies have highlighted a protective effect of PUFAs on brain tissue, due to the antioxidant/anti-inflammatory action on the chronic low grade inflammation substrate generated by MetS. However, studies on the preventative role of PUFAs on MCI and development of MetS are currently lacking.

## The role of probiotics on MetS

Probiotics are live microorganisms that have demonstrated beneficial effects on human health, affecting multiples pathways and axes.^78^ Major sources of probiotics are yogurt and kefir. Due to strong evidence that probiotics have anti-inflammatory properties, they could emerge as valuable tool for ameliorating MetS outcomes.

Treatment with probiotics has been demonstrated to significantly reduce mean arterial pressure and fasting plasma glucose, proving effectiveness in every MetS domain.^132^ Moreover, subjects treated with probiotics showed a significant decrease in high-sensitivity C-reactive protein (hs-CRP) and TNF-α levels compared to the control group, strengthening the hypothesis that probiotics may also improve MetS due to their anti-inflammatory effects.^11^^,^^14^^,^^133^

Some RCTs have been performed in elderly populations, more than ten of which, however, showed confounders in the selection of the population (for example: T2DM subjects, or non-standardized interventions, such as “Effects of kefir or milk supplementation”^134^).

Cicero et al.^76^ found a reduction in prevalence of MetS, cardiovascular risk factors and markers of IR in elderly patients through the 2-month period of treatment with a formula of *Lactiplantibacillus plantarum* PBS067, *Lactobacillus acidophilus* PBS066, and *Lactobacillus* *reuteri* PBS072 with active prebiotics. Specifically, the treatment group showed a statistically significant improvement in waist circumference and in fasting plasma insulin, total cholesterol, HDL cholesterol, non-HDL-C, TG, low-density lipoprotein (LDL)-C. The exclusive effect on the plasma lipid profile was analyzed in a meta-analysis of 13 RCTs conducted in patients with high, borderline and normal cholesterol.^77^ As a result, the use of probiotics appeared to significantly lower total cholesterol and LDL, although with no impact on HDL and TG. This effect was observed in all cohorts of subjects, and lipid profile at the baseline did not influence the results (Table 2). This finding is important as it may open a path for the possible use of probiotics in the prevention of hypercholesterolemia.

## The role of probiotics on MCI

Emerging clinical and experimental evidence has highlighted a bidirectional relationship between the gut microbiota and the brain, known as the microbiota-gut-brain axis (MGB).^135^ Countless of pathways are involved in this interconnection and a balanced composition of microbes is required for its optimal functioning. In fact, activation of gut inflammation, due to dysbiosis, is believed to be a cofactor in many pathological conditions; specifically, the microbiota has been proposed as a key player in the pathogenesis of both neurodegeneration and MetS.^136^ Therefore, restoring homeostasis of gut microbiota could be a reasonable goal for medical therapy in MCI, for which a medical therapy still needs to be codified.

To this end many efforts have been made to analyze the composition of microbiota in patients with neurodegenerative disorders. The most characteristic alterations in gut microbiota flora observed in patients with neurodegenerative disorders is the decrease in anti-inflammatory bacterial species such as *Bifidobacterium breve strain A1* and increase in pro-inflammatory bacterial species such as *Firmicutes* and *Bacteroidetes*.^78^^,^^137^ *Lactiplantibacillus plantarum* has also been studied for its anti-inflammatory properties, due to its high IL-10-inducing activity in immune cells.^82^^,^^138^

Different studies with animal models,^139^^,^^140^ some RTCs,^79^^,^^80^^,^^81^^,^^141^ and an open label single arm study^83^ have explored the possibility of restoring a correct proportion of microbiota as a potential treatment for MCI, by administering probiotic *B. breve strain A1* supplements in mice or patients diagnosed with MCI. These RCTs have tested similar amount of *B. breve* colony-forming unit (CFU) comparing this treatment to placebo for different time lapse, such as *B. breve* MCC1274, 2 × 1,010 CFU vs. placebo for 24 weeks,^79^ *B. breve* A1, 2 × 1,010 CFU vs. placebo for 16 weeks,^80^ *B. breve* MCC1274, 2 × 1,010 CFU) or placebo for 16 weeks,^81^ and two capsules daily approximately >2.0 × 1,010 CFU *B. breve* A1 for 12 weeks vs. placebo.^141^

A meta-analysis run in 2020 by Den. et al.^78^ revealed a significant difference in cognitive improvement between subjects with MCI or AD with and without probiotic supplementation. Indeed, subjects treated with probiotics showed better cognitive function after supplementation. The authors hypothesized that probiotics exert their effects by decreasing the level of inflammatory and oxidative effectors, such as malondialdehyde (MDA) and hs-CRP, as these have been found to be significantly decreased in the probiotics versus control group (Table 2).

However, no significant differences were observed at the level of total GSH, NO and total antioxidant capacity (TAC).^78^ Although a relationship of causality needs to be demonstrated, it is reasonable to note that immune signaling may not only be just a consequence of neurodegeneration, but also a key player in its process. As a matter of fact, increasing evidence demonstrates the relevance of inflammation in the progression of neurodegeneration.^142^^,^^143^^,^^144^^,^^145^ It has been reported that individuals with higher levels of inflammatory proteins in the blood, decades before the typical age of dementia symptom onset, are at increased risk for developing neurodegenerative disease.^145^

In studies on the effects of probiotics as a dietary supplement in subjects with MCI, results were consistent with an improvement in the composite score of attention and working/verbal memory. However, these studies present some limitations since the small population, the short observational period and the limited selection of cognitive domains assessed limit the reliability of their results. Therefore, increasing the sample size and improving the design of the trial could help clarify the effect of probiotics on general brain function.

## The role of probiotics in the prevention of MCI/MetS

Regarding the role of probiotics in the prevention of MCI, an interesting example of *in utero* use of probiotics to ameliorate memory decline in later life was reported by Zhang et al.^146^ Using animal models of lead-induced spatial memory deficit, maternal supplementation with *Lacticaseibacillus rhamnosus* at a dose of 109 CFU/day has proven its potential to mitigate lead-induced memory deficits. Notably, this result was independent of lead-induced alterations, as lead levels were measured and were not significantly reduced after treatment. Furthermore, *in vitro* experiments have revealed that *L. rhamnosus* inhibits growth of *Escherichia coli*, a pathogen involved in memory decline.^147^ Further research is needed to elucidate the mechanisms underlying probiotic-mediated memory protection and its potential for broader application in cognitive impairment prevention.^146^

Even with growing evidence from animal models supporting the use of therapies targeting gut microbiota before birth, to protect against the development of MetS, their effects on pregnant women are still unknown and these results require further research.^148^ However, animal models offer a valid example of how environmental factors during pregnancy and lactation exert an influence on adulthood development of MCI and MetS.^146^^,^^149^ With this awareness, various prevention strategies in early life have been explored: as an example, as reviewed by Hsu et al., supplementation with *Lactobacillus casei* during gestation in MetS induced rodent models, has been shown to benefit hypertension in adult progenies.^150^

The use of probiotics in the prevention of cognitive decline and metabolic features was also studied by Davari et al.,^151^ who aimed to evaluate the effect of probiotics on plasma glucose and insulin levels, behavioral tasks, and synaptic plasticity in a diabetic rat model. The probiotics solution was made by a mixture (each 334 mg) of *L. acidophilus* (American type culture collection [ATCC] 4356, 10ˆ10 CFU/g), *Bifidobacterium lactis* (Dutch chemical company [DSM] 10140, 10ˆ10 CFU/g), and *Lactobacillus fermentum* (ATCC 9338, 10ˆ10 CFU/g) and was administered for 8 weeks. It was effective in mitigating oxidative stress and inflammation and alleviating diabetic complications. Indeed, results showed that supplementation with a probiotics solution (a mixture of *L. acidophilus*, *B. lactis*, and *L. fermentum*) significantly improved spatial memory and task learning capabilities in diabetic animals. Moreover, electrophysiological recordings revealed enhanced synaptic transmission compared to the control group; biochemical measurements showed mitigated oxidative stress, as proven by increased level of superoxide dismutase activity and reduced level of 8-hydroxydesoxyguanosin production. Interestingly, no significant effects were observed on behavior, synaptic plasticity, or biochemical markers in control rats, suggesting that the potential therapeutic role of probiotics is specifically on diabetes-related oxidative stress and neurodegeneration.

Mo et al.^152^ explored the effects of probiotic supplementation in overweight human subjects, showing how probiotics can positively influence some anthropometric parameters of MetS in borderline-healthy patients. *Lactobacillus curvatus* HY7601 and *Lactobacillus plantarum* KY1032 were administered orally for 12 weeks, resulting in a reduction in visceral fat mass and waist circumference, along with an increase in adiponectin levels. This positive impact could be triggered by a modulation of gut microbiota, presented here as a key determinant for activation of specific signaling pathways in obese humans. This RCT^152^ suggests how probiotics could be used as a promising strategy for the prevention of obesity and related metabolic disorders (Table 2).

In conclusion, we can say that studies on the effect of probiotics MetS and MCI show contrasting results. However, given the lack of RCTs with standardized outcome in the literature, more research is needed in order to clarify whether these nutraceuticals can be validated as codified treatment for both MetS and MCI.

## Conclusions

The study of nutraceuticals is an emerging field of growing interest due to the necessity for strategies in the prevention and treatment of MetS and MCI. These conditions are strongly connected, and both are a prelude to more severe outcomes. Since MetS and MCI share pathophysiological pathways, the keys of which are inflammation and oxidative stress, substances with antioxidant and anti-inflammatory properties have been explored in healthy and impaired models, with heterogeneous results. While there is sufficient literature on the biochemical effects of some nutraceuticals, there is still limited evidence for the positive effects of vitamin E, PUFAs, probiotics and resveratrol on subjects with impaired cognitive function or MetS and weak evidence supporting the intervention with these nutraceuticals as prevention. The literature lacks studies with standardized interventions, clear and codified cognitive and metabolic assessments, and specific pathognomonic biomarkers of MetS and MCI. Also, considering the long-term requirement of medications when it comes to MetS and MCI, longer trials and observational studies in this area are needed. In particular, we stress the need for RCTs with more rigorous endpoints and more specific markers that can provide reliable evidence to assess the adequacy of interventions with nutraceuticals.

## Limitations of the study

Firstly, the narrative character of this study represent itself a limitation. As a matter of fact, the unsystematic selection, lacked formal metanalytic method to derive the most trustable RCTs from the existing literature, could have produced a biased study selection. Secondary, we have not organized our review by mechanisms of actions or biological origin of components, which represents another flaw. Finally, the preclinical character of MCI and MetS represents an obstacle for rigorous identification of population and outcome, leading us to include a variety of studies that are often too different from each other to be reproducible.

## Declaration of interests

The authors declare no competing interests.
